# Where to Be Sick

**Published:** 1873-06

**Authors:** 


### WHERE TO BE SICK.

At home, by all means, unless there are peculiar circumstances. To be sick at a hotel or a boarding-house is dreadful to think of; and if, in addition, it is five hundred or a thousand miles or more away from home, with all the depressing influences of such a situation, it is no wonder that we read of so many invalids dying in distant parts of the country and across the great sea. The death of a New Yorker was announced the other day in Nice, on the shores of the Mediterranean, in his seventy-fifth year. He had owned and occupied one of the most elegant mansions in Fifth Avenue; the appointments within were almost regal; and yet he died in a little room, more than three thousand miles away, among strangers and a strange people.

No person should go from home for health who has not plenty of time and money. To have a limited amount of means, and to see them slowly melting away day after day, even with a moderate improvement, is enough to worry a sensitive mind to death. And it drives many thousands to death every year; puts them into their graves long before their time.

To be benefited by a change of climate in any ailment which has been a long time present, necessarily requires months and months of time; it should be a residence, rather than a visit. Those most likely to be benefited by going from home are such as are able to go about and to attend to business every day, but who are evidently invalids as a result of worry and anxiety and responsibility and care or family trouble arising from death or social causes. Absence and travel will always break in upon depressing trains of thought and help them out of the ruts of sameness and mechanical routine.

A person thin in flesh, easily chilled, easily worried, with any kind of daily cough and a pulse always over ninety, should never think of going farther than a day's journey from home.

